# Editorial: Future trends and directions of using mHealth strategies to prevent and treat cardiovascular diseases

**DOI:** 10.3389/fpubh.2023.1246918

**Published:** 2023-07-07

**Authors:** Zhao Ni, Santi Martini, Erin M. Spaulding, Arief Hargono, Seth Shay Martin

**Affiliations:** ^1^School of Nursing, Yale University, New Haven, CT, United States; ^2^Yale Institute for Global Health, Yale University, New Haven, CT, United States; ^3^Faculty of Public Health, Airlangga University, Surabaya, Indonesia; ^4^School of Nursing, Johns Hopkins University, Baltimore, MD, United States; ^5^The Johns Hopkins Hospital, Johns Hopkins Medicine, Baltimore, MD, United States

**Keywords:** mobile health, mHealth, cardiovascular diseases, strategies, trends, prevention, innovation

Preventing and treating cardiovascular diseases is of paramount importance, given their status as the leading cause of death worldwide (1). Unlike other diseases that are disproportionately influenced by social-demographic, ethnic, and geopolitical factors, cardiovascular diseases prevail as the primary cause of death across sexes and racial/ethnic groups in both developing and developed countries (1). For example, in the United States, cardiovascular diseases account for 20% (2–4) of all deaths and cost about $239.9 billion yearly (4, 5). Tragically, every 33 s a person succumbs to cardiovascular diseases (2, 4). In China, cardiovascular diseases cause nearly 4 million deaths annually (6) and impose a substantial economic burden on society (7, 8) due to heightened healthcare expenditures and productivity losses. Consequently, innovations in preventing and treating cardiovascular diseases hold immense significance on a global scale.

Mobile health, commonly known as mHealth, refers to the use of portable electronic devices with software applications to deliver health services and manage patient information (8–11). Studies have demonstrated the potential of mHealth in preventing cardiovascular diseases and supporting cardiovascular rehabilitation (8, 12–16). By providing personalized and real-time motivation and care, mHealth caters to the needs of people at high risk of developing cardiovascular diseases or those living with such conditions. Our proposed Research Topic, titled “*Future trends and directions of using mHealth strategies to prevent and treat cardiovascular diseases*” aims to capture the current status of mHealth strategies for cardiovascular diseases prevention and treatment in real clinical and community settings. We received a total of seven manuscripts and accepted four of them, which collectively provided valuable insights into current mHealth limitations and future directions.

Firstly, while the effectiveness of leveraging mHealth to promote cardiovascular health has been established, there is a scarcity of research focused on developing metrics to assess engagement with mHealth interventions as well as their feasibility, acceptability, and usability. A paper from Germany found that existing studies often employ self-defined questionnaires, followed by usage logs and interviews, as the primary tools for measuring these criteria. However, the validity and reliability of these self-defined instruments are often not established. There is a lack of recognized standards for evaluating mobile apps for cardiovascular diseases, primarily due to the complexity of cardiovascular diseases, which involve diverse risk factors and patient health needs. Although some guidelines have been issued for evaluating digital health interventions, such as the World Health Organization's guidelines titled “*Recommendations on Digital Interventions for Health System Strengthening*” (17), there is still a need for greater adoption and tailoring of such standards for mHealth studies in cardiovascular diseases. To address this gap, further research is needed to create more standardized evaluation instruments that can effectively measure the impact of mHealth interventions.

Secondly, another notable limitation in current research on mHealth is the inadequate reporting of the design and development process of mHealth interventions. While many studies focus on reporting outcomes, few provide details on their design and development. This gap hinders the adoption of mHealth interventions in the field of cardiovascular diseases. Studies from Australia and China emphasize the need for reporting the intricate design and development details of mHealth interventions. Furthermore, they discuss the importance of using a user-centered design approach in the development and implementation of mHealth interventions for cardiovascular diseases by involving key stakeholders, such as patients, healthcare professionals, and family members. We anticipate that future mHealth interventions will integrate new technologies, including artificial intelligence, machine learning, deep learning, virtual reality, augmented reality, and chatbot technology with traditional mobile technologies such as smartphone applications, wearables, web browsers/dashboards, and text messaging. These advancements aim to enhance the effectiveness of mHealth interventions and promote their capabilities to address individual concerns within the complex real-world environment. Researchers should consider including cost-effectiveness analysis as an important criterion to measure the effectiveness of mHealth interventions, in addition to traditional health outcomes such as blood pressure, readmission rates, and mortality. By incorporating such analysis, researchers enable policymakers and healthcare professionals to make informed decisions regarding the benefits of mHealth interventions. This, in turn, may propel the implementation of mHealth interventions in real-world settings.

Additionally, infodemiology has emerged as a prominent area of interest in mHealth research. As people commonly search for information online to prevent and treat cardiovascular diseases, harnessing the potential of infodemiology has become crucial to understanding how people access and engage with health information online. Researchers can gain valuable insight into users' behaviors, information gaps, and potential avenues for improving the dissemination of accurate and reliable information. A study in Malaysia developed an online platform to record the information patients search for and empower the public to appraise topic-specific health information. Future research should explore methods to incorporate infodemiology into mHealth interventions to empower individuals to make informed decisions and establish a framework for tailored interventions to address specific informational needs.

In conclusion, this Research Topic aims to address limitations and explore the current status of mHealth strategies in real clinical and community settings. By examining effectiveness, design and development, integration of new technologies, and infodemiology, we aim to contribute to the advancement and implementation of mHealth interventions for cardiovascular diseases.

## Author contributions

ZN drafted the initial version of the manuscript. All authors contributed to the article and approved the submitted version.
